# Effective Cleaving Parameters for Multimode Gradient Index CYTOP Polymer Fiber

**DOI:** 10.3390/polym12112491

**Published:** 2020-10-27

**Authors:** Ivan Chapalo, Antreas Theodosiou, Georgii Pobegalov, Sergei Chapalo, Kyriacos Kalli, Oleg Kotov

**Affiliations:** 1 Institute of Physics, Nanotechnology and Telecommunications, Peter the Great St. Petersburg Polytechnic University, 29, Polytechnicheskaya st., 195251 St. Petersburg, Russia; george.pobegalov@nanobio.spbstu.ru (G.P.); kotov@rphf.spbstu.ru (O.K.); 2 Electromagnetism and Telecom Department, University of Mons, 31 Boulevard Dolez, 7000 Mons, Belgium; 3 Photonics and Optical Sensors Research Laboratory (PhOSLab), Cyprus University of Technology, Nicolaou Saripolou 33, 3036 Limassol, Cyprus; theodosiou.antreas@gmail.com (A.T.); kyriacos.kalli@cut.ac.cy (K.K.); 4 Institute of Civil Engineering, Peter the Great St. Petersburg Polytechnic University, 29, Polytechnicheskaya st., 195251 St. Petersburg, Russia; chapalo.se@edu.spbstu.ru

**Keywords:** CYTOP, polymer optical fiber, fiber cleave, multimode fiber, fiber optics

## Abstract

We experimentally address simple, low-cost and effective methods for the cleaving of multimode CYTOP optical fibers using razor blades. The quality of fiber end-face preparation depends on various parameters. The necessity of the near-field intensity pattern inspection for adequate evaluation of cleaved fiber end-faces is demonstrated. Razor blades of different manufacturers are evaluated for manual cleaving, as well as automated cleaving with controlled speed and temperature. The cleaving technique with both slowed motion of the razor blade and increased temperature up to 90 °C demonstrated the best quality of fiber end-faces. Typical cleaving defects are highlighted, whereas the cleave quality was characterized in terms of the light intensity profile emitted by the fiber in near field.

## 1. Introduction

Polymer optical fiber (POF) draws significant interest from researchers and industry due to its flexibility, ease of use, low Young modulus, and safety in biomedicine [1,2,3]. Among POFs, the CYTOP fiber has radically low attenuation in the telecom transparency windows and high bandwidth that makes it the best candidate for fiber-to-the-home communication technologies; it has also been used extensively for research into optical fiber sensing [1,4,5]. Applications based on fiber Bragg gratings [6,7,8,9], intermodal interference [10], single-mode-multimode-single-mode structures [11], cladding micro waveguides [12] and Brillouin scattering [13] have been investigated.

In general, POFs are low-cost solutions compared to their silica fiber counterparts, since they do not require expensive termination equipment and delicate preparation methods; their large cores and highly multimode and plastic nature means that the cleaving process of POFs can be made simply using a low-cost razor blade. However, in the case of CYTOP fiber, which also has a multimode core but with a gradient-index profile, certain tasks require very good quality of fiber end-faces to provide both controllable launching conditions and spatial intensity distribution measurements. These tasks may include core refractive index profile evaluation [14,15], modal power distribution measurement utilizing intensity profile at the multimode fiber (MMF) output facet [16,17], differential mode delay and bandwidth investigation and optimization using restricted mode launch [18,19,20]. In addition, the investigation and operation of intermodal fiber interferometers (IFIs) (or specklegram sensors) require controlled launch and undistorted speckle pattern, in particular for IFI property manipulation that is dependent on launching conditions [21] and the coordinates of photodetectors at the fiber cross-section [22]. Furthermore, a good-quality end-face leads to a more precise and low-loss fiber interconnections that can enhance the transmission characteristics of fiber optic links.

There are several studies that relate to cleaving POFs and, in particular, microstructure fiber designs where the integrity of air holes is important after the cleaving process. For this reason, a variety of fiber end-face preparation techniques were explored including simple cut by razor blade [23], polishing [24,25], low-temperature cleaving [26,27], focused ion-beam milling [27,28], semiconductor dicing saw and ultraviolet laser cleaving [28]. They were applied not only for polymethyl methacrylate (PMMA) materials but also, for example, cyclic olefin copolymer (TOPAS) fibers [28,29].

The razor blades (RB) approach is the most common and simple method for which different aspects of RB cleaving have been investigated, such as cleaving temperature [27] and speed of cleave [30]; indeed, portable cleavers have been proposed and tested [31]. It was demonstrated that proper blade temperature was required so as not to exceed the glass transition temperature of the material thereby significantly improving the cleaving quality [27,29]. Articles [30,31] show that fiber heating could be replaced by having blade motion with slow speed, due to the temperature–time equivalence in polymers. In addition, it was highlighted that the blade-motion component perpendicular to cleaving direction helped to reduce “crazing” on fiber facet surface.

In recent years, significant polymer-sensing applications were developed using multimode CYTOP gradient index fibers, and the focus of this work aimed to investigate the optimum cleaving conditions for particular POFs using the RB method. We demonstrate that near-field intensity pattern (NFIP) inspection is a necessary procedure for evaluation of the cleaving quality along with the microscope inspection of the cleaved fiber facet. RB cleaving quality is evaluated for a sample of different commercially available razor blades. A novel setup based on a motorized rotation stage was assembled, tested, and applied in experiments. The influence of cleaving speed and temperature was explored, and it was found that the best results can be achieved when both increased temperature and slow motion of the blade are applied. Different types of cleave defects are also demonstrated.

## 2. Experimental Setup

GigaPOF-50SR (Chromis Fiberoptics (Warren, NJ, USA)) fiber was utilized in experiments. The following methods of RB cleaving were investigated: manual cleave, cleave with controlled speed, cleave with controlled temperature, and their combinations. Firstly, we performed simple manual cleaves of POF using RBs from different manufacturers (a sample of 6 RB models produced by Bic (Clichy, France), Derby (Tuzla, Turkey), Feather (Osaka, Japan), Gillette (Boston, MA, USA), Sputnik (St. Petersburg, Russia), and Wilkinson (London, UK)) and a surgical scalpel produced by B. Braun (Melsungen, Germany). Experiments with each RB model were performed multiple times, ensuring that the given part of the blade was used for cleave only once.

**Figure 1:**
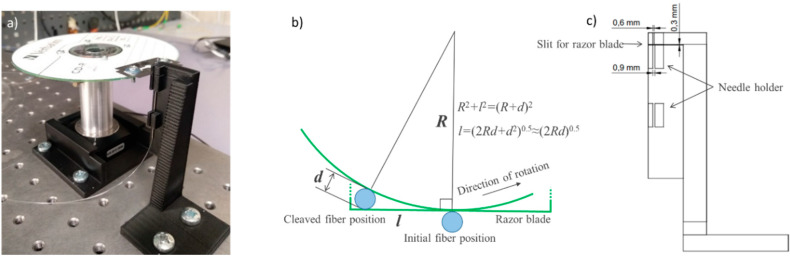
(**a**) A photograph of the cleaving setup based on the motorized rotational stage; (**b**) a schematic of the cleaving geometry calculation; (**c**) a drawing of the fiber holder stage.

In order to investigate possibilities to improve cleaving quality and stability, we assembled a setup based on motorized rotational stage (Newport Agilis AG-PR100 (Irvine, CA, USA)), which enabled us to perform cleaving with a controlled speed (Figure 1a). The stage was fixed horizontally using specially designed and 3D-printed holders. As demonstrated in [30], improved cleaving quality can be achieved if the blade has a significant crosswise component of movement with respect to the direction of cleave. Following this recommendation, we faced the blade tangentially to the direction of its rotation. At that, the length *l* of the RB involved in cleaving can be estimated as *l* ≈ (2·*d*·*R*)^0.5^, where *d* is the fiber diameter and *R* is the blade’s distance from the axis of rotation (Figure 1b). To increase the blade’s crosswise movement component, we fixed the razor significantly far from the axis of rotation using a standard CD disc with a diameter of 120 mm mounted on a duralumin cylinder screwed into the rotation stage. The length of the blade involved in cleaving was estimated to be 7.8 mm. To slightly increase this value, we turned the blade to a small angle so that the front edge of the razor (relatively to the direction of blade motion) had a larger distance from the axis of rotation than the rear edge.

To hold the fiber, we utilized a hypodermic needle of standard G21 with an inner diameter of 500 µm (the outer diameter of the GigaPOF fiber was 490 µm); the needle-syringe plastic connector was removed beforehand. The needle was fixed on a specially designed and 3D-printed vertical stage which contained a horizontal 0.3 mm slit (resolution limit of the utilized 3D printer) for the RB (Figure 1c); the end of the needle was located in close proximity to the slit. Hence, utilizing the rotational stage and fixing the fiber at the vertical stage, we could perform reproducible RB cleaving with controlled speed, whilst enabling tangential component of the motion.

The motorized stage provided three levels of rotation rate, which were calculated to be 0.008, 0.19 and 3.6 degrees/s. The tangential velocity of the razor was estimated to be 8 µm/s, 210 µm/s and 4 mm/s, correspondingly. During cleaving, due to the resistance of the fiber material, the real speed of rotation was slightly decreased depending on the razor blade model and cleaving temperature. The averaged cleaving time was measured to be 14 min, 28 s and 2 s, correspondingly with the levels of rotation rate.

In addition to different RB models and cleaving rates, we provided the possibility of performing cleaving at different temperatures. To avoid the complexity of the setup we did not control the heating of the RB and the fiber separately; we simply utilized a household hair dryer located at a proper distance from the setup to provide approximate necessary temperature that was calibrated in advance. This enabled us to perform cleaving at room temperature, as well as at temperatures up to 100 °C.

To evaluate the cleaving quality, we recorded microscope images of a cleaved facet using 10× and 40× microscope objectives. This helped us to qualitatively evaluate the appearance of the cleave, such as the flatness and the shape of a cross-section. Here, in fact, the quality of polycarbonate protection cladding the surface was mainly evaluated. Additionally, we recorded NFIPs when incoherent light was launched into the fiber. The LED of a smartphone, 20× microscope objective and USB web camera Logitech c270 (Lausanne, Switzerland) were used for this purpose. The NFIPs were utilized as an indicator of the cleaved CYTOP core region quality. A clean gradient intensity profile proved a successful cleave, whereas the presence of roughness and irregularities indicated that the cleave was not good enough.

**Figure 2:**
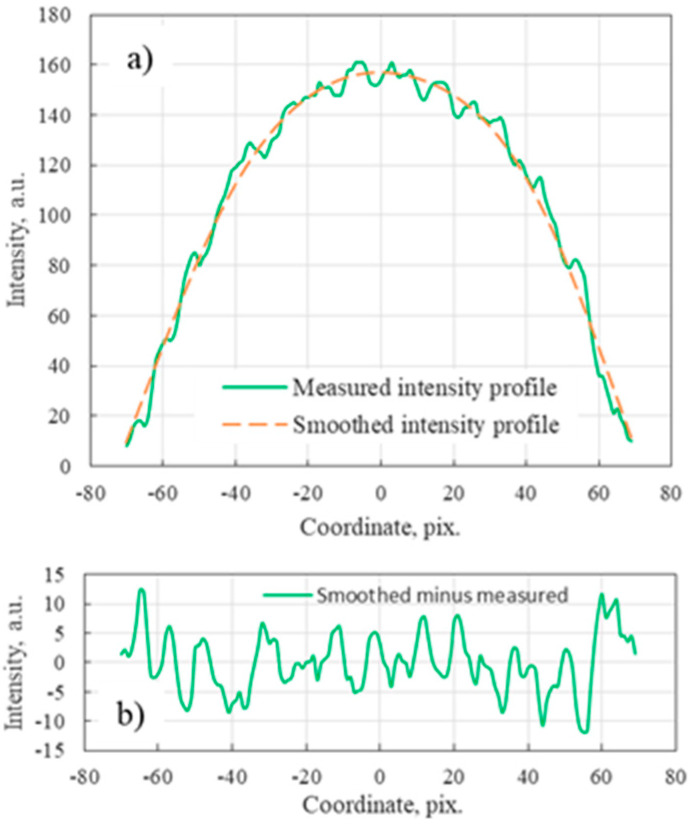
Illustration of the irregularity coefficient calculation: (**a**) original and smoothed intensity distribution in the NFIP cross section and (**b**) their difference.

To evaluate cleaving quality numerically, we introduced the parameter of irregularity *K_I_* according to the formula

(1) $$K_{I}=\sqrt{\frac{\sum_{i=1}^{N}{\left(x_{i}-x_{i}^{s}\right)}^{2}}{N}}\cdot \frac{100\%}{\bar{x_{}^{s}}}$$

where *x_i_* is an intensity distribution over a chosen cross-section of the NFIP, $x_{i}^{s}$ is a smoothed intensity distribution over a chosen cross-section of the NFIP, *i* is a pixel number at the NFIP cross-section, *N* is a number of pixels at the NFIP cross-section and $\bar{x_{}^{s}}$ is a mean value of smoothed NFIP cross-section. The algorithm of the *K_I_* calculation is illustrated in Figure 2. The intensity distribution over a chosen cross-section of the NFIP was smoothed (Figure 2a), and the smoothed intensity distribution was subtracted from the initial intensity distribution (Figure 2b). The irregularity coefficient was obtained by calculating the standard deviation of the difference, normalizing it by the mean value of smoothed intensity distribution and multiplying by 100%. To avoid complexity of processing algorithms, we calculated the *K_I_* for two mutually perpendicular cross-sections of the NFIP and utilized the maximum of these two values.

## 3. Experimental Results and Discussion

**Figure 3:**
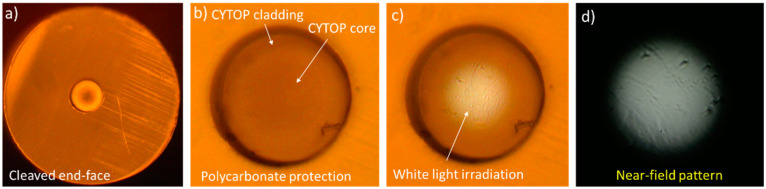
Comparison of microscopic image (**a**–**c**) and near-field pattern (**d**) end-face inspection methods: the near-field profile pattern demonstrates defects (**d**) that can be missed by the microscopic image (**b**). (**a**) cleaved end-face, (**b**) CYTOP core-cladding region, (**c**) CYTOP core-cladding region when white light was launched into the fiber, (**d**) near-field intensity pattern.

CYTOP fiber is a multimode graded-index fiber; therefore it is important to provide enough smoothness of its end-face, especially at its core region. We have found that unlike microstructured POFs, for which cleave quality is usually evaluated by inspection of an air hole’s structural integrity by viewing a microscopic image of a fiber facet, a microscopic image of the CYTOP fiber facet does not always provide an adequate representation of the cleave quality. To demonstrate this, a set of microscopic images of particular cleaved fiber facets and the NFIP when light from an incoherent source was launched into the fiber are presented in Figure 3: the image of the cleaved end-face (Figure 3a), the image of the CYTOP core-cladding region (Figure 3b), the same but when white light was launched into the fiber (Figure 3c), and the NFIP (Figure 3d). It is obvious that despite a respectively clean image of the core region of the cleaved end-face (Figure 3b), the near-field intensity distribution indicates a significantly disturbed surface (Figure 3d). Hence, in this work, to adequately evaluate the cleaving quality, we analyzed NFIP along with microscopic images of the cleaved facet. Smooth parabola-shaped near-field profile distribution indicated a successful cleave.

**Figure 4:**
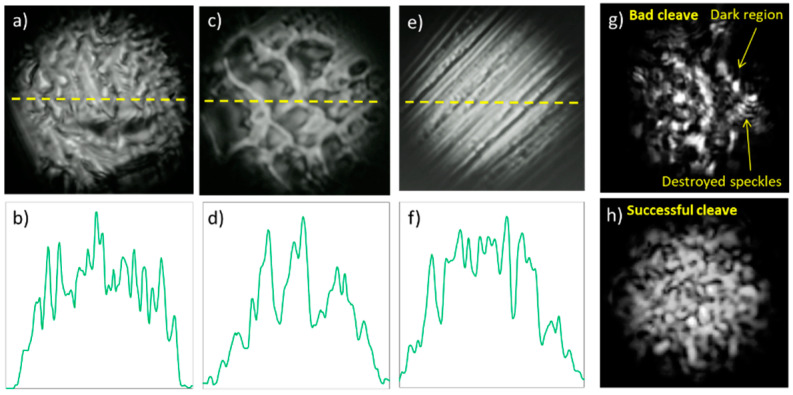
Three examples of poor-quality cleaves of the CYTOP fiber: near-field patterns, (**a**,**c**,**e**) and intensity profiles, (**b**,**d**,**f**) generated by incoherent fiber launch. The dashed line indicates the location of the near-field profile at the near-field pattern. Comparison of speckle patterns formed at poor (**g**) and successful (**h**) cleaved end-faces when the fiber was launched using a coherent source.

For reference, in Figure 4a–f we demonstrate examples of a bad-quality cleave and corresponding horizontal intensity profiles (obtained from the core center). The cleaves were obtained using the manual razor blade cleaving technique. It can be seen that the intensity profiles are almost destroyed (Figure 4b,d,f). In addition, Figure 4g,h compares near-field speckle patterns recorded for poor- (Figure 4g) and good-quality (Figure 4h) surfaces of the CYTOP fiber facets. It is obvious that a poor cleave results in degradation of the speckle pattern appearance: decomposition of the speckles to smaller components and the presence of dark regions.

The investigation of the CYTOP fiber cleaving was performed as follows: manual cleaving exploring different RB models and selection of appropriate RBs, cleaving with different speed, and cleaving at different temperature and speed.

### 3.1. Manual Razor Blade Cleave

**Figure 5:**
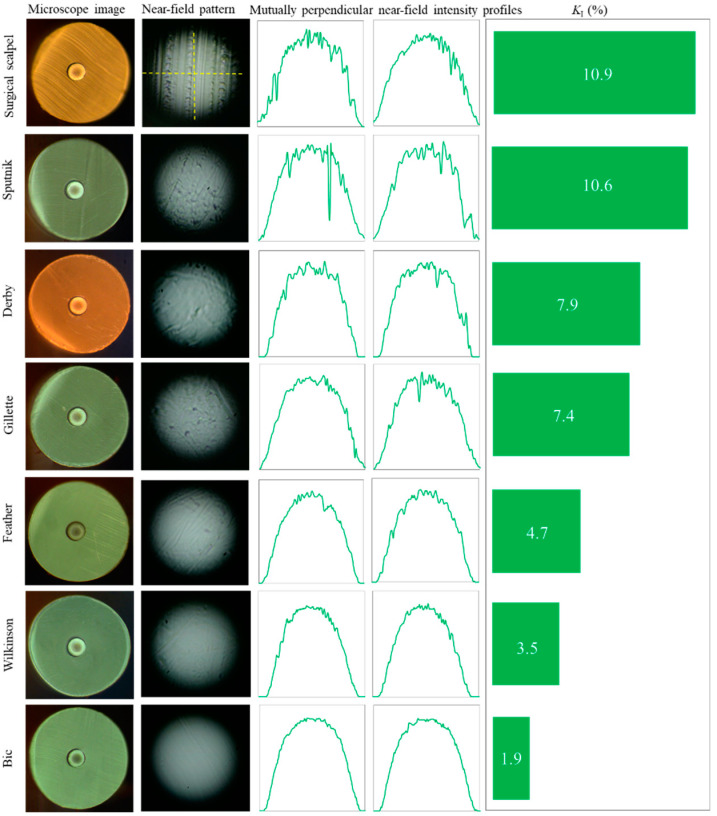
Typical successful cleaves obtained by different razor blade models. Dashed line indicates the location of the near-field profile at the near-field pattern.

Typical microscope images, NFIPs, near-field intensity profiles (two mutually perpendicular profiles for each NFIP) of successful cleaves and the parameter of irregularity *K_I_* for each blade model are presented in Figure 5. It can be seen that cleave quality (and, in particular, quality of NFIP) is far from ideal and significantly depends on the blade’s model. For our sample of razor blades, taking into account the photographs of NFIPs (column 2 in Figure 5) in conjunction with horizontal and vertical profile graphs (columns 3–4 in Figure 5) and irregularity coefficients, the best intensity profiles were obtained by razor blades Bic (*K_I_* = 1.9%), Wilkinson (*K_I_* = 3.5%) and Feather (*K_I_* = 4.7%). The surgical scalpel demonstrated the worst results (*K_I_* = 10.9%), most likely because of its higher thickness and insufficient sharpness in comparison with razor blades.

**Figure 6:**
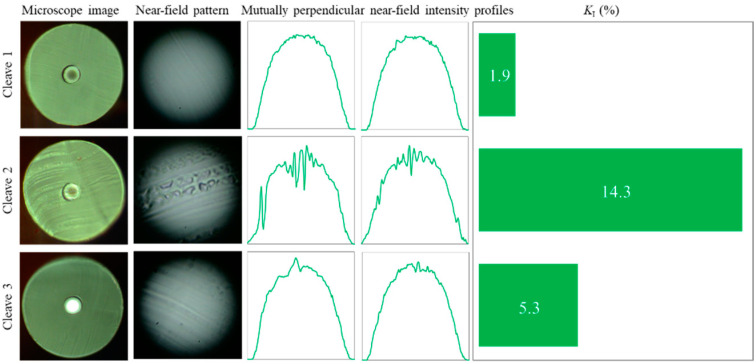
Three consecutive cleaves (obtained by Bic razor blade): end-face quality is not stable cleave by cleave.

It is important to note, that multiple cleaving using even “successful” RB models did not always demonstrate stable (reproducible) results (Figure 6); consecutive cleaves randomly provided very different quality: *K_I_*^1^ = 1.9, *K_I_*^2^ = 14.3 and *K_I_*^3^ = 5.3 (in the experiments, we ensured that every cleaving part of the blade was used only once). It should be mentioned that cleaved fiber facets had a typical bevel at one of their edges; however, this did not affect the NFIP quality, as it occurred in the polycarbonate protection cladding.

Thus, it can be concluded that manually performed cleaving can produce sufficiently good end-faces (depending on the task); however, the razor blade model should be carefully selected. With this, even properly selected RBs do not guarantee reproducible high-accuracy cleaves. Therefore, in situations when cleaving quality is critical, each cleave has to be inspected by microscope imaging and by observing near-field intensity profiles to ensure sufficient quality.

### 3.2. Cleave with Controlled Speed

The automated setup based on a motorized rotational stage (Figure 1) enabled control of the cleaving speed and direction. Three possible values of the rotation speed provided by the stage were investigated for POF cleaving. We tested Bic, Wilkinson and Feather razor blades selected in the previous section. The last two razor blade models were successfully applied, while the Bic razor blade cleaves were unsuccessful: the razor blade got stuck during the cleaving process and the stage stopped the rotation. Possible reasons for this could be the higher thickness of the Bic blades compared to the others (we measured it as 0.11–0.12 mm versus 0.1 mm for the other blades) and, probably, the different profile and/or angle of the blade edge.

**Figure 7:**
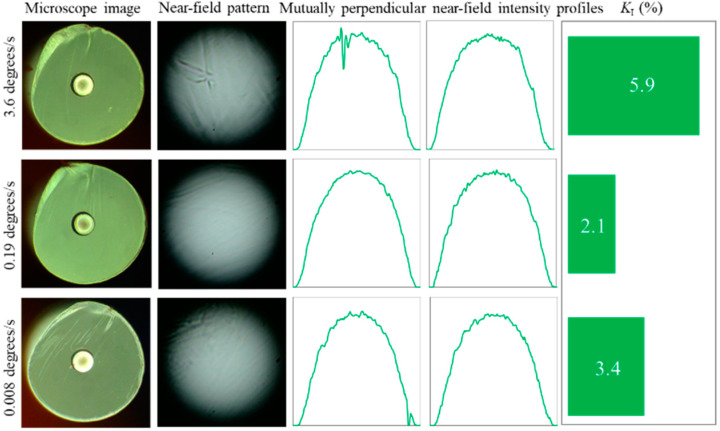
Typical results of cleaves performed at different speeds with the Feather razor blade (RB). The cleaving time was 2 s, 28 s and 14 min (from top to bottom).

Figure 7 demonstrates the cleaving results obtained using the Feather RB. In general, the ability to control the speed of the razor blade movement improved cleaving quality (in the CYTOP region) and reproducibility of the results. At that, medium (28 s for one cleave) speed of rotation improved cleaving quality compared to the fastest (2 s per cleave) speed (*K_I_* = 2.1% versus *K_I_* = 5.9%), which corresponds to the results presented in [30]. However, the slowest (14 min per cleave) rotation speed did not demonstrate significant improvement of the cleave’s quality (*K_I_* = 3.4%), and was very time consuming. Thus, making sure of the absence of significant effect, we excluded the slowest rotation speed of cleaving from further experiments. It should be mentioned that the quality and the shape of the end-faces in this case was even worse than in the case of manual cleave.

**Figure 8:**
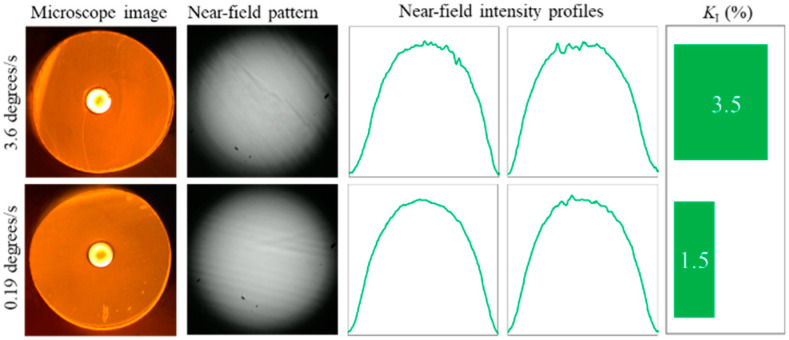
Typical results of cleaves performed at two different speeds with the Wilkinson RB. Cleaving time was 2 s and 28 s. The slowest speed (14 min per cleave) was excluded from the experiments.

To investigate the possibility of increasing cleaving quality, we conducted the aforementioned experiment utilizing the Wilkinson RB (Figure 8), which demonstrated better cleaving quality of manual cleave in comparison with the Feather RB (*K_I_* = 3.5% versus *K_I_* = 4.7%). The results demonstrated a slight improvement of the NFIP quality, especially at a high speed of RB motion (3.6 degrees/s): *K_I_* = 3.5% versus *K_I_* = 5.9% at 3.6 degrees/s and *K_I_* = 1.5% versus *K_I_* = 2.1% at 0.19 degrees/s. The quality and the shape of the end-faces were increased as well. Taking this into account, we conducted temperature experiments utilizing the Wilkinson RB.

### 3.3. Cleave with Controlled Speed and Temperature

**Figure 9:**
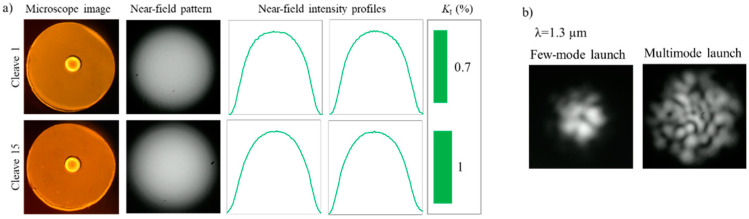
(**a**) Cleaves performed at temperature ≈ 90 °C and rotation speed of 0.19 degrees/s (28 s per cleave) using the Wilkinson RB. Two examples (cleave no. 1 and cleave no. 15) of a series of 20 consecutive cleaves performed using the same part of the same RB. (**b**) Near-field images of the speckle patterns obtained using distributed feedback (DFB) laser with λ = 1.3 µm and a single-mode pigtail utilizing central and offset launch.

In order to investigate cleaving at temperatures close to the glass transition temperature of the material as recommended in literature [23], we investigated cleaves at temperatures from 70 to 100 °C with the step of 10 °C (the glass transition temperature of the CYTOP material is 108 °C [32]). The rotation speed of 0.19 degrees/s (28 s per cleave) was utilized according to results reported in the previous section. The results of the experiments demonstrated essential improvements in the cleaving quality and stability (reproducibility); the optimal temperature was found to be at proximity of 80–90 °C (Figure 9). It can be seen that the intensity profile had a very smooth graded-index shape (*K_I_* = 0.7%). Moreover, it was found that the same blade region can be utilized not once but several times without significant degradation of the cleaving quality. We performed 20 consecutive cleaves by the same blade region, and at least the first 15 cleaves did not demonstrate significant degradation of the cleave quality, namely the irregularity parameter increased from *K_I_* = 0.7% for the first cleave up to *K_I_* = 1% for the 15^th^ cleave. Figure 9b demonstrates near-field speckle patterns when the POF was excited by the coherent light source (λ = 1.3 µm) in underfilled and multimode regimes. It is obvious that speckle patterns have proper view (shape) and do not demonstrate any degradation unlike the example in Figure 4g; they demonstrate good ability to control launching conditions.

**Figure 10:**
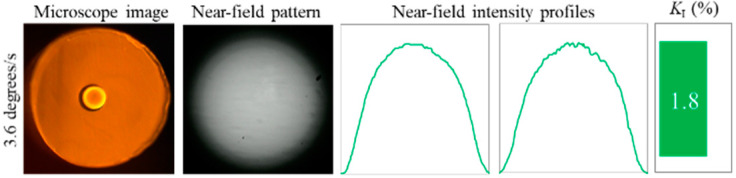
Cleave performed at temperature ≈ 90 °C and rotation speed of 3.6 degrees/s (2 s per cleave) using the Wilkinson RB.

In addition to the obtained results, we investigated cleaves that were performed at the same temperature but with rotation speed of 3.6 degrees/s (2 s per cleave) (Figure 10). The results demonstrated slight degradation of NFIP (*K_I_* = 1.8%) but more distorted flatness of the end-face compared to previous rotation speed. Nevertheless, it can be concluded that the quality of the NFIP is still good enough.

### 3.4. Cleaving Defects

**Figure 11:**
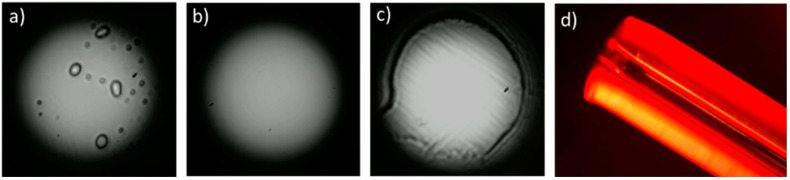
Demonstration of different kinds of the near-field intensity pattern imperfections: (**a**) presence of cleaving product (dust and pieces of material) (**b**) that can be removed by end-face cleaning; (**c**) exfoliation in the proximity of the core-cladding boundary; (**d**) the CYTOP core-cladding structure shift in relation to the protective cladding.

During the experiments, we separated typical cleaving defects into three main groups: crazing, grooves and cleaving product. The first group can be observed as random POF facet imperfections: Figure 4a,c is a bright examples of that. The second group demonstrates a set of groves oriented along the direction of the blade movement. It can be observed in Figure 4e, Figure 5 (surgical scalpel) and Figure 6 (cleave 2). It is likely related to the razor blades’ quality. The third group has the apparent defects, namely the presence of dust and pieces of material after the cleaving process. They appears at the NFIP as separated dots or circles and can be removed by a series of lint-free wipes and gentle cleaning with alcohol (Figure 11a,b).

Additionally, in some cases when the fiber was subjected to higher temperature (around 100 °C) or lower temperature but longer exposure time, some impairment of the NFIP and the fiber structure was observed near the core-cladding boundary (Figure 11c). Probably, it is a result of the core separating from the cladding. Additionally, in some cases microscope observations of the fiber from the side surface caused the suspicion of the CYTOP core-cladding structure shift in relation to the protective cladding (Figure 11d). This correlates with microscopic images of the fiber end-faces where certain CYTOP structure shift can also be observed (see for example, Figure 10).

## 4. Conclusions

In this article, we presented the investigation of the CYTOP fiber cleaving using the razor blade technique. We demonstrated the importance of the near-field intensity profile inspection as a main instrument for cleaving quality evaluation along with the microscope end-face inspection. To evaluate the cleaving quality numerically, we introduced the irregularity parameter, which assesses a normalized standard deviation of the near-field intensity profile from its smoothed representation.

Such techniques as manual cleave, cleave with controlled speed and cleave with controlled temperature were investigated. It was found that manual cleaving significantly depends on the RB type (*K_I_* can vary from ≈ 2% up to ≈ 10% for successful cleaves), and even appropriate razor blades can provide very different results from cleave to cleave. In general, proper selection of the RB gives quite good cleaves; however, the process should be accompanied by microscope imaging and NFIP inspection of the fiber end-faces.

Cleaving with controlled speed improves the quality of the NFIP by approximately two times and additionally increases stability (reproducibility) of the results. However, performing controlled cleaving together with proper temperature radically improves the quality of the NFIP (*K_I_* value reduced by five times from 3.5% down to 0.7% for chosen RB). In addition, this regime enables multiple cleaving (up to 15–20 cleaves) without significant quality degradation utilizing the same RB.
